# Construction and analysis of gene co-expression network in the pathogenic fungus *Ustilago maydis*

**DOI:** 10.3389/fmicb.2022.1048694

**Published:** 2022-12-07

**Authors:** Cinthia V. Soberanes-Gutiérrez, Alfredo Castillo-Jiménez, Ernesto Pérez-Rueda, Edgardo Galán-Vásquez

**Affiliations:** ^1^Laboratorio de Ciencias Agrogenómicas, de la Escuela Nacional de Estudios Superiores Unidad León, Universidad Nacional Autónoma de México, León, Guanajuato, Mexico; ^2^Licenciatura en Biología, Facultad de Ciencias, Universidad Nacional Autónoma de México, Ciudad Universitaria, Mexico City, Mexico; ^3^Unidad Académica Yucatán, Instituto de Investigaciones en Matemáticas Aplicadas y en Sistemas, Universidad Nacional Autónoma de México, Mérida, Mexico; ^4^Departamento de Ingeniería de Sistemas Computacionales y Automatización, Instituto de Investigación en Matemáticas Aplicadas y en Sistemas. Universidad Nacional Autónoma de México, Ciudad Universitaria, Mexico City, Mexico

**Keywords:** *Ustilago maydis*, gene co-expression network, WGCNA, gene expression, virulence genes, transcription factors

## Abstract

**Introduction:**

Biological systems respond to environmental disturbances and a wide range of compounds through complex gene interaction networks. The enormous growth of experimental information obtained using large-scale genomic techniques such as microarrays and RNA sequencing led to the construction of a wide variety of gene co-expression networks in recent years. These networks allow the discovery of clusters of co-expressed genes that potentially work in the same process linking them to biological processes often of interest to industrial, medicinal, and academic research.

**Methods:**

In this study, we built the gene co-expression network of Ustilago maydis from the gene expression data of 168 samples belonging to 19 series, which correspond to the GPL3681 platform deposited in the NCBI using WGCNA software. This network was analyzed to identify clusters of co-expressed genes, gene hubs and Gene Ontology terms. Additionally, we identified relevant modules through a hypergeometric approach based on a predicted set of transcription factors and virulence genes.

**Results and Discussion:**

We identified 13 modules in the gene co-expression network of U. maydis. The TFs enriched in the modules of interest belong to the superfamilies of Nucleic acid-binding proteins, Winged helix DNA-binding, and Zn2/Cys6 DNA-binding. On the other hand, the modules enriched with virulence genes were classified into diseases related to corn smut, Invasive candidiasis, among others. Finally, a large number of hypothetical, a large number of hypothetical genes were identified as highly co-expressed with virulence genes, making them possible experimental targets.

## Introduction

Organisms are complex entities with controlled gene expression that respond to intracellular and extracellular signals. A significant number of studies using high-throughput technologies, such as microarrays, and RNA sequencing (RNA-seq), have been conducted in recent years to investigate differential gene expression, enabling the detection of co-expressed genes in a specific condition (Kitano, 2002; Trewavas, 2006; Costa-Silva et al., 2017; Stark et al., 2019). In this context, gene co-expression networks (GCNs) are used to systematic study the gene expression in an organism, where the network is made up of a set of nodes that represent the genes and a set of edges that represent significant co-expression relationships (Stuart et al., 2003; Junker and Schreiber, 2008; van Dam et al., 2018). These networks exhibit a scale-free topology, which means there are a few strongly connected nodes, and a large number of nodes (or genes) with few connections (van Noort et al., 2004; Tsaparas et al., 2006). GCNs allows the identification of clusters and modules of genes with similar expression patterns across multiple experimental conditions; indeed, these modules are frequently enriched in genes with comparable biological functions (Zhang and Horvath, 2005; Mueller et al., 2017; van Dam et al., 2018). The co-expression relationships are assessed through a similarity metric, such as correlation, which is calculated for each pair of genes through a pattern of gene expression between various phenotypes (van Dam et al., 2018).

*Ustilago maydis* is a Basidiomycota fungus (Basse and Steinberg, 2004), a biotrophic pathogen of corn (*Zea mays*) and its probable ancestor Teosinte (*Zea mays* ssp. *parviglumis* and ssp. *mexicana*; Agrios, 2005), which causes corn smut or Huitlacoche. *Ustilago maydis* is characterized by dimorphism from yeast to filaments, which occurs during its life cycle or as a response to nutritional elements, and pH (Horst et al., 2012; Rodríguez-Kessler et al., 2012; Martínez-Soto and Ruiz-Herrera, 2013; Olicón-Hernández et al., 2019; Ferris and Walbot, 2020). The life cycle of *U. maydis* can be divided into distinct phases that are tightly linked to plant colonization. Teliospore germination results in the formation of haploid basidiospores which divide by budding to form unicellular and saprophytic haploid cells. Compatible, non-pathogenic haploids *a1b1* and *a2b2* fuse to form a dikaryon, where cell recognition and fusion are regulated by a pheromone/receptor system that is encoded by mating-type loci. The dikaryon proliferates, branches, and penetrates the plant via the formation of appressoria. The cells switch to filamentous growth if they are heterozygous for the *b* locus. The *b* locus encodes the two homeodomain transcription factors *bE* and *bW*. These dikaryotic hyphae invade the plant and lead to the characteristic symptoms of the disease: chlorosis, formation of anthocyanins, the development of galls into the aerial parts of the plant (in which diploid teliospores are accumulated). Teliospores germinate outside the host and produce phragmobasidia, which give rise to budding basidiospores, reinitiating the cycle (Brefort et al., 2009; Saville et al., 2012; Snetselaar and McCann, 2017).

The large amount of information associated to this fungus, makes *U. maydis* an excellent model to study biotrophic pathogens and sexual fungal development; in addition to its haploid stage in which the fungus forms sporidia that divide by budding allowing the use of conventional microbiology techniques. In this regard, the fungus allows the ability to construct diploids in the laboratory, as well as for DNA recombination and vesicular transport, because of its facility of transformation with exogenous DNA. Furthermore, its sensitivity to genomic segments replaced by mutated gene copies by homologous recombination; the possibility of *in vitro* analysis of the dimorphic transition (yeast to mycelium), and the fact that its genome is completely sequenced (Kämper, 2004; Bakkeren et al., 2008; Dean et al., 2012; Soberanes-Gutiérrez et al., 2015; Schuster et al., 2016). For instance, global analysis, as microarray-based and RNA-seq transcriptome data have identified organ-specific expression patterns, cell types, and stages of the effector gene repertoire in *U. maydis* (Skibbe et al., 2010; Lanver et al., 2018; Matei et al., 2018).

In this study, a gene co-expression network for *U. maydis* based on a Weighted correlation network analysis was conducted. To do this, expression data belonging to 168 samples belonging to 19 series, which correspond to the GPL3681 platform deposited in the NCBI, were considered. Then, this network was clustered into modules that were posteriorly analyzed by using gene ontology and KEGG annotations. Finally, those transcription factors and virulence related genes were identified to determine its functional role in the context of the co-expression network.

## Data and methods

### Datasets

**Figure 1:**
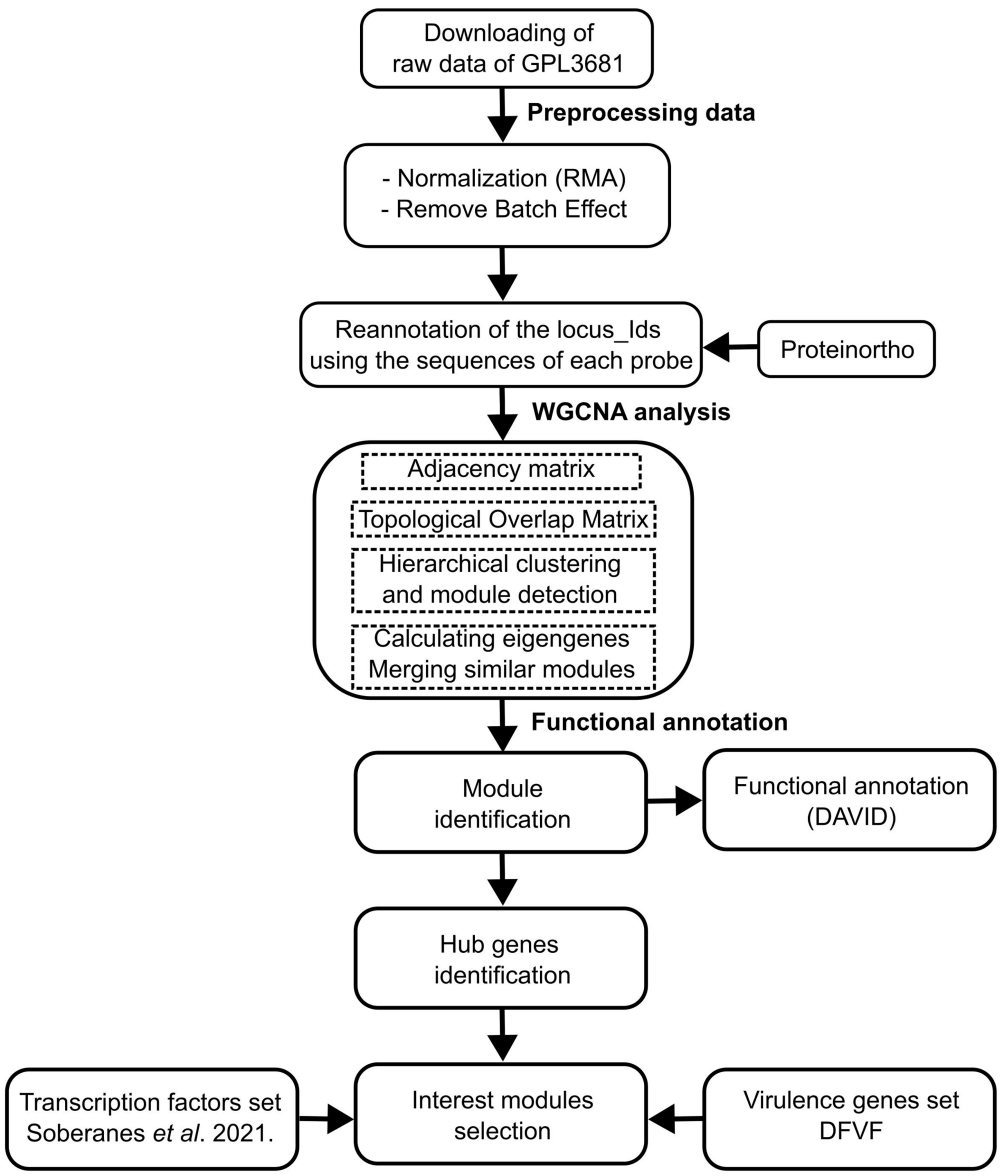
Schematic workflow of the data of preparation, processing, and analysis.

The gene expression compendia for *U. maydis*, were obtained from the Gene Expression Omnibus (GEO) database. We used raw data of 168 samples of 19 series from Affymetrix array (ID: GPL3681), that contains experiments related to biotrophic development (Vollmeister et al., 2009; Heimel et al., 2010; Lanver et al., 2014; Scherer et al., 2016), regulation of morphology and dimorphism (Vollmeister et al., 2009; Elías-Villalobos et al., 2011; Wang et al., 2011), genes required for virulence (Eichhorn et al., 2006; Molina and Kahmann, 2007; Teertstra et al., 2011; Wang et al., 2011; Schuler et al., 2015), and pathogenic development (Heimel et al., 2010A; Supplementary Table S1). The Limma Bioconductor packages in R were used to preprocess the expression data, which included normalization using the Robust Multichip Average (RMA) technique (Ritchie et al., 2015). Then, a Bach adjustment was applied using the function removeBatchEffect with default parameters (Ritchie et al., 2015). Finally, a matrix was constructed containing genes in rows, and samples in columns. See Figure 1 (section: Preprocessing data) and Supplementary Table S2.

### Construction of gene co-expression networks

The gene co-expression networks were constructed using the Weighted correlation network analysis (WGCNA) software (Langfelder and Holvarth, 2008). The scale-free topological features of biological networks were added using the pickSoftThreshold function with a power (*β*) value of 14. Then, an adjacency matrix was constructed, using signed correlation networks, where nodes with negative correlation were regarded as disconnected, as well as the pairwise biweight midcorrelation coefficients. This approach was selected because it outperforms the Spearman and Pearson correlation methods (Song et al., 2012; Bakhtiarizadeh et al., 2018). In a posterior step, the adjacency matrix was converted into a Topological Overlap Matrix (TOM), with a higher TOM value, allowing for the identification of gene modules for each pair of genes with strong interconnectivity. Signed correlation networks, pairwise biweight midcorrelation coefficients, and β value were posteriorly used.

The genes were clustered into modules with similar expression patterns, with an average linkage hierarchical clustering technique (flashClust function), and using the cutreeDynamic function. A 1-TOM as a distance matrix with a minimum module size of 20, was considered. Therefore, the modules with high connected eigengenes were combined (mergeCloseModules function, minimum height of 0.25). Each module was assigned with a color (Horvath, 2011). See Figure 1 (section WGCNA analysis) and Supplementary Table S3.

To determine the effect of hubs on the modules, they were exported using the exportNetworkToCytoscape function, and the top 400 connections in each module were selected. Then, the degree of connectivity for each node (*K*), which is defined as the number of edges adjacent to each node, was estimated (Junker and Schreiber, 2008). See Figure 1 (section: functional annotation).

### Distribution of TFs and virulence genes

The identification of TFs was achieved by scanning protein sequences of whole proteomes using InterProScan (v5.25–64.0), and hmmsearch (v3.1b2), by default parameters, and selecting those proteins with domains associated to TFs as anticipated by Soberanes-Gutiérrez et al. (2021). An incidence rate for the genome, and a heatmap for each genome were calculated to identify and quantify each dataset.

Additionally, a total of 2058 proteins associated with virulence were obtained from the Database of virulence factors in fungal diseases, DFVF (Lu et al., 2012). The DFVF provides information of 2058 pathogenic genes associated with 228 fungal strains from 85 taxa. We identified the virulence protein-encoding genes in the gene co-expression network of *U. maydis* using the program ProteinOrtho (v 6.0.15), with an *E-value* of 1e-05, a sequence coverage of 50%, and minimal percent identity of best blast hits of 25%, except for the report of singleton genes without any hit (Lechner et al., 2011). See Figure 1 (section: functional annotation) and Supplementary Table S4.

### Functional annotation analysis

To identify the biological process in each module, we used the Database for Annotation, Visualization, and Integrated Discovery (DAVID^1^), which is a gene functional classification system that integrates a set of functional annotation tools (Huang et al., 2009). See Figure 1 (section: functional annotation).

### Enrichment analysis

To evaluate the functional association of TFs and virulence genes into the modules, an enrichment analysis using a hypergeometric test was conducted. The resulting distribution describes the probability of finding *x* domains associated with a particular category in a list of interest *k*, from a set of *N* domains containing *m* domains that are associated with the same category. We set statistical significance at a *p*-value of less than 0.05. All analyses were performed in Python.^2^ See Figure 1 (section: functional annotation) and Supplementary pipeline S1.

## Results and discussion

### Construction of gene co-expression network

**Figure 2:**
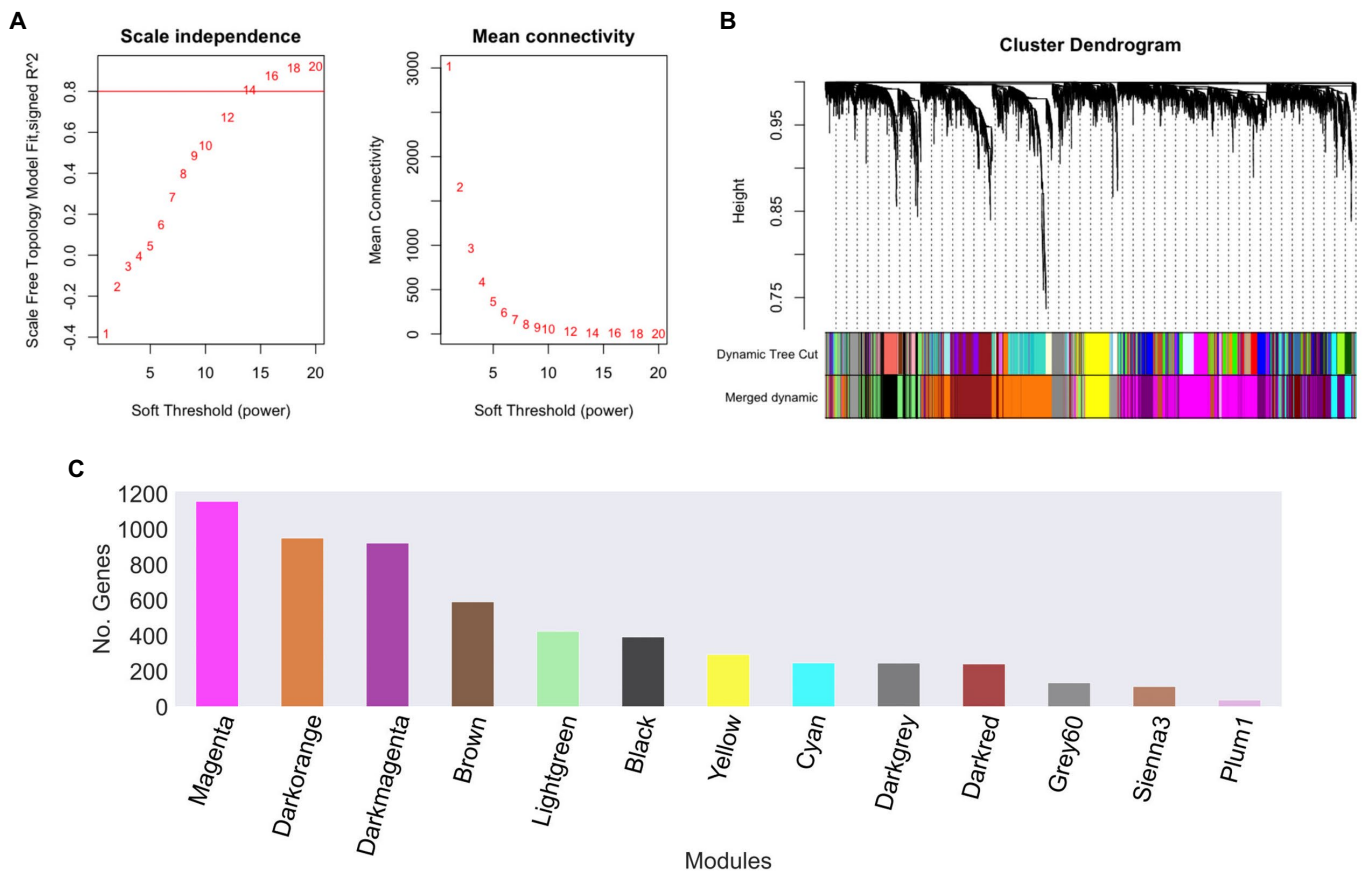
*Ustilago maydis* co-expression modules. **(A)** Scale independence and mean connectivity of network topology for different soft-thresholding, **(B)** A module hierarchical clustering tree was constructed based on the correlation of the amount of expression among genes. Each color corresponds to the genes belonging to the same module in the cluster tree, **(C)** The number of genes in each module. The grey module was removed because it is reserved to genes that are not part of any module.

In order to explore the relationships between different gene datasets of *U. maydis*, we used a gene expression compendium obtained from the GEO database. These samples were used to construct a gene co-expression network using WGCNA software, with a soft threshold power *β* of 14 as the scale-free topology criterion, since it resulted in the lowest power reaching the greatest value of the *R*^2^ = 0.8 (Figure 2A; Langfelder and Holvarth, 2008).

To reconstruct the network, we considered signed networks, because WGCNA considers the sign of the underlying correlation coefficient, and that has been shown that it can identify modules with more significant enrichment of functional groups (Medina and Lubovac-Pilav, 2016; Liu et al., 2018). The network includes a total of 5,766 predicted open reading frames (ORFs), i.e., a coverage of 85.23% of the ORFs. From this, 13 modules were identified (see Figure 2B), being, the largest one with 1,158 genes (Magenta), whereas the smallest module contains 39 genes (Plum1; Figure 2C; Table 1).

**Table 1:** Co-expression modules of *Ustilago maydis*.

Module	Size (number of genes)	Total of TFs	Total of virulence genes	Functional terms [GO (BP^a^)]	Metabolic pathways
					(KEGG^b^)
Black	349	41	29	Energy derivation by oxidation of organic compounds	Metabolic pathways
Brown	592	22	56	Carbohydrate metabolic process	-
Cyan	248	22	19	Cell cycle	Cell cycle- yeast
Darkgrey	247	13	17	Signal transduction	Protein processing in endoplasmic reticulum
Darkmagenta	923	46	43	Nucleobase-containing compound metabolic process	DNA replication
Darkorange	951	71	44	Ribonucleoprotein Complex Biogenesis	Ribosome
Darkred	242	18	10	-	Spliceosome
Grey60	136	3	6	Cellular amino acid metabolic process	Biosynthesis of amino acids
Lightgreen	426	24	25	Organic acid metabolic process	Metabolic pathways
Magenta	1,158	44	91	Transport	Endocytosis
Plum1	39	6	0	Cellular catabolic process	Proteasome
Sienna3	115	2	12	Intracellular signal transduction	Amino sugar and nucleotide sugar metabolism
Yellow	295	11	26	Transport	Autophagy-other

a The most enriched Biological process term.

b The most enriched KEGG term.

### Biological function of co-expressed modules

**Figure 3:**
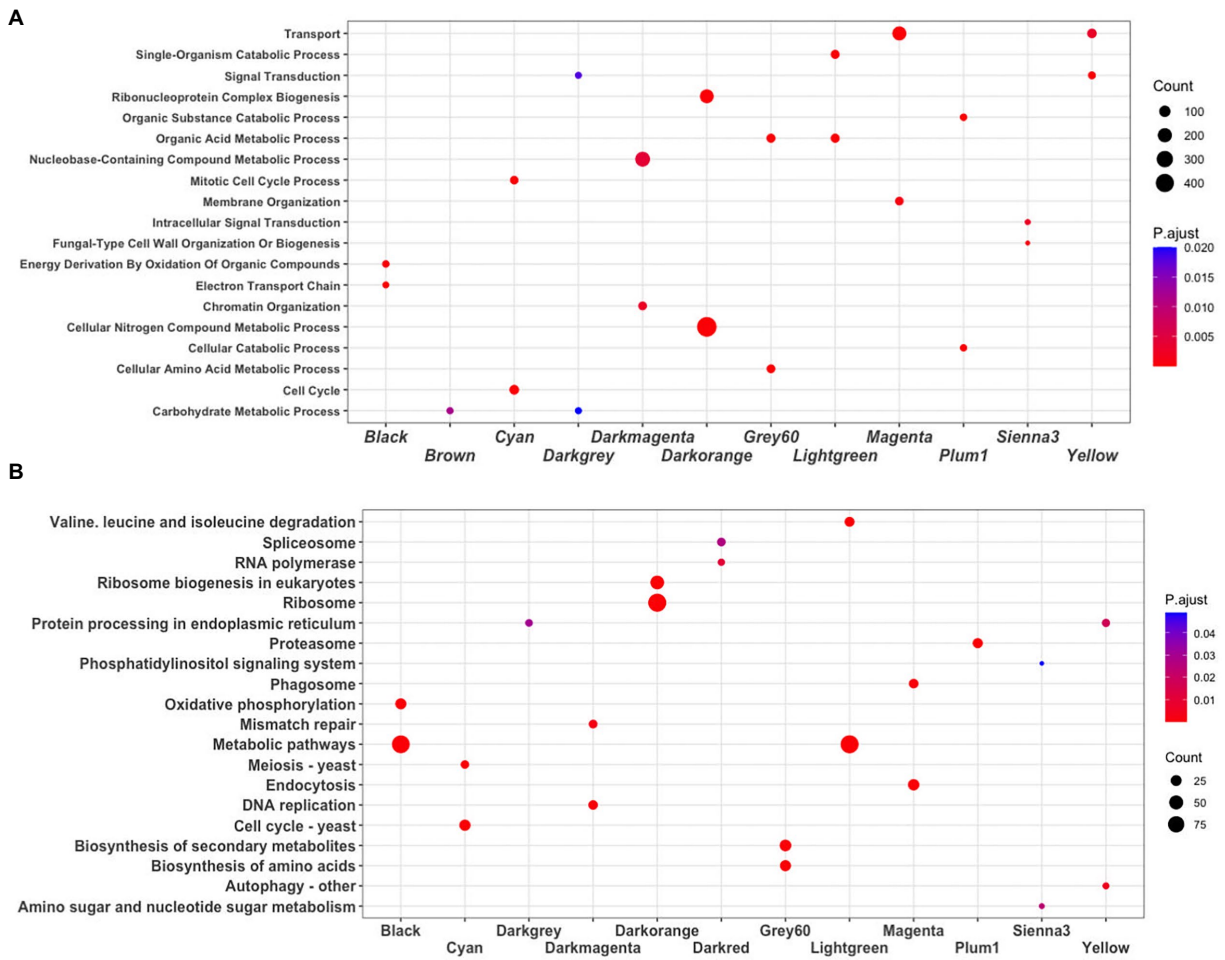
Enrichment analysis. **(A)** Biological process GO terms, and **(B)** KEGG pathways. The dot plot shows the main terms (FDR < 0.05) identified using DAVID. The size of a dot represents the number of genes associated with each term, and the color of dots represents the *p*-adjusted values.

To identify the main functions represented in the modules, they were analyzed in terms of their Gene Ontology and KEGG assignments. In the following, we described the most relevant modules. The Darkorange module contains 951 genes, representing 16.49% of the total genes of the network. From these, 71 TFs and 44 proteins probably related to virulence processes were identified. In functional terms, the biological process Ribonucleoproteins Complex Biogenesis was the most enriched function (GO:0022613, FDR = 7.3e-78; Figure 3A), mainly associated with the Ribosomal process (KEGG, FDR = 5.5e-35; Figure 3B). These processes result in the production of component macromolecules, as well as, the assembly and organization of RNA-protein complex constituents, such as the 18S rRNA pseudouridine methyltransferase (UMAG_06195), 25S rRNA (adenine645-N1)-methyltransferase (UMAG_10715), and ribosomal 40S subunit proteins, among others.

The Cyan module contains 248 genes, representing 4.30% of the total number of genes of the network. From these, 22 TFs and 19 virulence proteins were identified. In functional terms, the Cell cycle was the most enriched function (GO:0007049, FDR = 1.2e-29; KEGG, FDR = 1.6e-19; Figure 3); where stands out a putative DNA topoisomerase II (UMAG_03501), the TSD2 protein related gene, required for DNA replication (UMAG_05770), putative tubulin beta chain (UMAG_10558), and Cyclin involved in mating-related cell cycle arrest (UMAG_03758).

Finally, the Yellow module comprises 295 genes, that represents 5.11% of the total of genes analyzed in this work. 11 TFs and 26 protein encoding genes associated with virulence were found. In functional terms, the signal transduction (GO:0007165, FDR = 2.4e-05; Figure 3A) and Autophagy (KEGG, FDR = 5.2e-03; Figure 3B), were the most enriched functions. This include phosphatidylinositol 3-kinase VPS34 (UMAG_00453) which is needed for cytoplasm to vacuole transport and autophagy (Takahashi et al., 2001); putative ubiquitin-like protein ATG8 (UMAG_05567) that is an important constituent of autophagosomes and cytoplasm to vacuole transport, mainly expressed in cell death (Nair et al., 2012; Soberanes-Gutiérrez et al., 2021); and TORC1 (UMAG_00801), which is also expressed in cell death (Soberanes-Gutiérrez et al., 2021).

### Hub genes in co-expressed modules

In order to evaluate the contribution of hub genes in each module, we screened them into the top 400 connections. The rationale behind this analysis is that hubs are the most connected nodes and are expected to play an important role in biological networks, as suggested by Langfelder et al. (2013) and Liu et al. (2018). Therefore, in the Darkorange module, where Ribonucleoprotein Complex Biogenesis related genes were identified, the gene UMAG_10469 (Small subunit ribosomal protein S5e), which is involved in translation initiation by recruiting elF2 and ElF3 factors to the 40S subunit (Lumsden et al., 2010; Sharifulin et al., 2013) was found as a hub, with a connectivity degree of 42, which means that it is co-expressed with 42 other genes inside its module. On the other hand, in the Darkmagenta module the MCM10-essential chromatin-associated protein involved in the initiation of DNA replication (UMAG_10135), and Putative DNA polymerase epsilon (UMAG_01008) with a degree of 39, were identified as hubs. These genes are related to the replication process, which agrees with functions found in the enriched analysis (Araki et al., 1992; Storfie and Saville, 2021). Moreover, another interesting hub is UMAG_05113 in the Grey60 module, it codes for Threonine dehydratase which is a key enzyme in BCAA synthesis, which catalyzes the conversion L-threonine into α-ketobutyrate, furthermore, this enzyme is inhibited by the end product of the metabolic pathway (Liang et al., 2021). Finally, in Sienna3, Brown, Cyan, and Magenta the hubs are UMAG_04807, UMAG_03644, UMAG_05198, and UMAG_10929, respectively, which code for hypothetical proteins (Table 2).

**Table 2:** Hub genes and their encoding proteins in the GCN modules of *U. maydis*.

Module	Hub gene (^a^)	Functional description
Darkorange	UMAG_10469 (42)	Probable 40S ribosomal protein S5
Lightgreen	UMAG_05230 (38)	Salicylate hydroxylase
	UMAG_05791 (38)	Hypothetical protein
Grey60	UMAG_05113 (37)	Threonine dehydratase
Yellow	UMAG_02843 (27)	Putative serine-type endopeptidase
Black	UMAG_10695 (34)	Putative NADH dehydrogenase (ubiquinone) 78 K chain precursor
Plum1	UMAG_10231 (32)	Proteasome regulatory subunit N12
	UMAG_05860 (32)	putative 20S proteasome subunit beta 7
Darkred	UMAG_00105 (28)	Iron transport multicopper oxidase
Magenta	UMAG_10929 (35)	Hypothetical protein
Darkmagenta	UMAG_10135 (39)	Related to MCM10 - essential chromatin-associated protein involved in the initiation of DNA replication
	UMAG_01008 (39)	Putative DNA polymerase epsilon, catalytic subunit A
Cyan	UMAG_05198 (35)	Hypothetical protein
Brown	UMAG_03644 (61)	Hypothetical protein
Darkgrey	UMAG_02752 (27)	Hypothetical protein
	UMAG_05528 (27)	Glycosyl hydrolase 53 domain-containing
	UMAG_12024 (27)	Homeodomain TF Hdp1
Sienna3	UMAG_04807 (26)	Hypothetical protein

a The degree node of each of the hubs is shown in parentheses.

### Highly enriched modules in TFs and virulence genes

To identify modules with a high abundance of TFs and virulence factors, these were mapped with proteinortho and functional annotations. From these, we identified that the Darkorange module contains the largest number of TFs with 71, while the Sienna3 is the module with the smaller number of TFs, with only two. This result is consistent with previous research on bacteria co-expression modules, in which TFs are distributed across the modules at different proportions and may be associated to regulate gene expression of the genes into the module (Sastry et al., 2019). On the other hand, the magenta module contains the largest number of virulence genes with 96, while the grey60 is the module with the smaller number of virulence genes, with six (Supplementary Table S5).

To identify if a module contains a greater amount of TFs or virulence genes than expected randomly, an enrichment analysis was performed using a hypergeometric test. We found that Plum1, Cyan, Darkorange and Black modules were enriched for TFs; while the Brown, Magenta and Yellow modules were enriched with virulence genes (Supplementary Figure S1). In this context, the black module was the most enriched module with TFs, and the brown module was the most enriched with virulence genes with -log(*p*-value) of 4,579 and 3,083, respectively.

#### Superfamilies associated with TFs

To evaluate if the modules with enriched TFs have a preference for a particular superfamily, the TFs were classified using the assignments of the Supfam database. Posteriorly, they were clustered hierarchically based on Euclidean distance measure and Ward’s methods for linkage analysis with the Z-scores of the frequency of the superfamily.

We determined that Nucleic acid-binding proteins superfamily (SSF50249) is most frequent in Cyan and Darkorange modules. This superfamily is constituted by 16 families where the most abundant in *U. maydis* is Cold shock DNA-binding. It is a protein domain of around 70 amino acids that helps the cell survive in temperatures above the optimum, probably by chromosomal condensation (Obokata et al., 1991). In fungi, these proteins are related to development, morphology, stress response and pathogenicity process (Sato, 1995; Fang and St. Leger, 2010; Kim, 2014).

**Figure 4:**
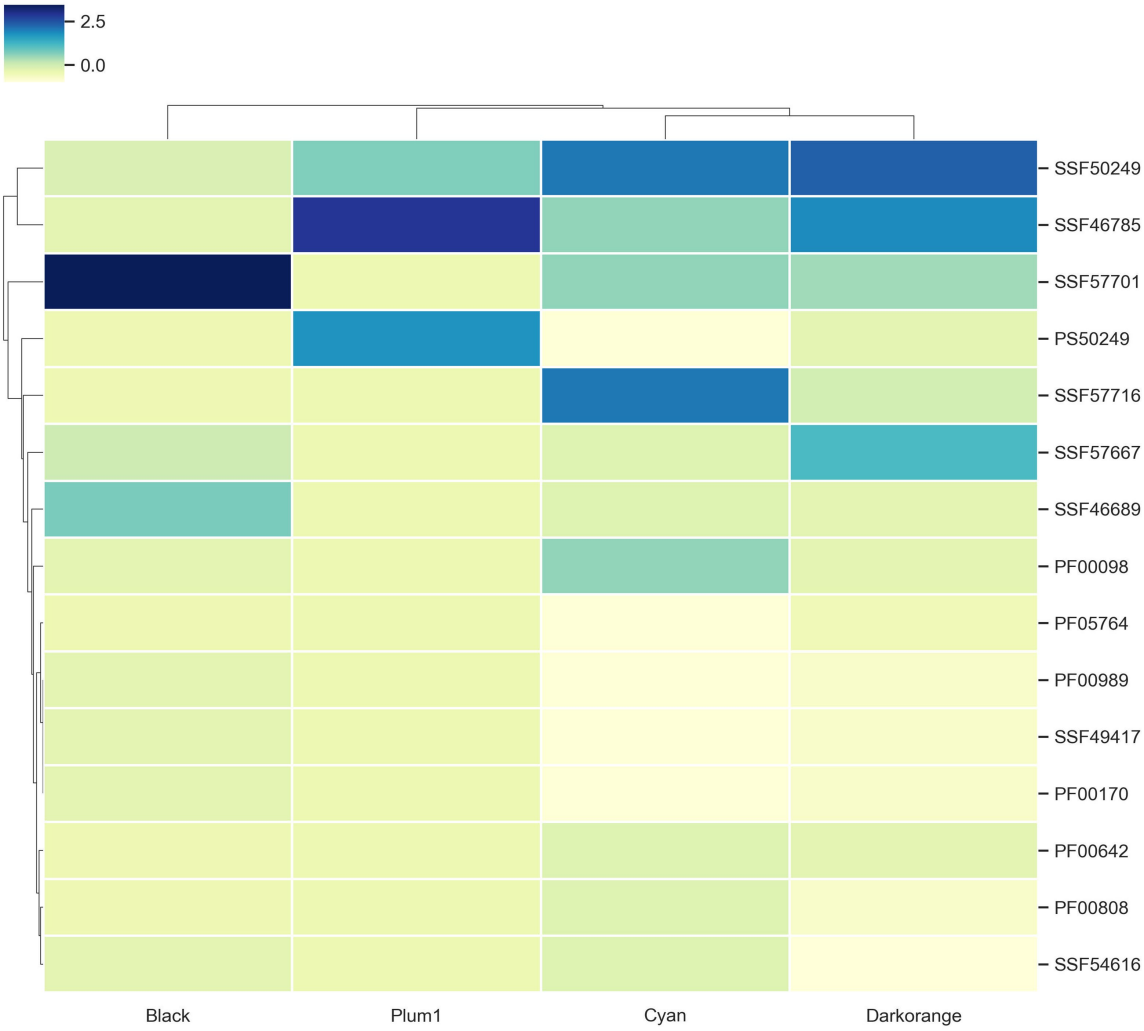
TF families identified as frequent in the enriched modules. Z-score hierarchical clustering based on Euclidean distance measure and Ward’s method for linkage analysis. Each row represents the superfamily ID.

Additionally, Winged helix DNA-binding domain (SSF46785) was found in Darkorange and Plum1 modules. This superfamily is found in widely distributed in all the organisms (Knox and Keller, 2015), and is related to multiple biological functions, including transcriptional repression, transcription factors involved in cell differentiation, and heat-shock transcription factors (Gajiwala and Burley, 2000; Figure 4). Heat shock factor (HSF)-type belongs to SSF46785 and plays a role in virulence of fungal pathogens (Veri et al., 2018).

Finally, the Zn2/Cys6 DNA-binding domain (SSF57701) is the most frequent in Black, Cyan and Darkorange. This is the largest superfamily of extant fungal-specific domains (Shelest, 2008), and is present in proteins that regulate a number of activities, including carbon and nitrogen metabolism, amino acid and vitamin synthesis, stress response, pleiotropic drug resistance, meiosis, and morphogenesis (MacPherson et al., 2006). In particular, UMAG_06256 (member of this superfamily) is involved in transcription from RNApol II promoter as a response of chemical stimulus (Cerqueira et al., 2014).

#### Virulence genes and disease

**Figure 5:**
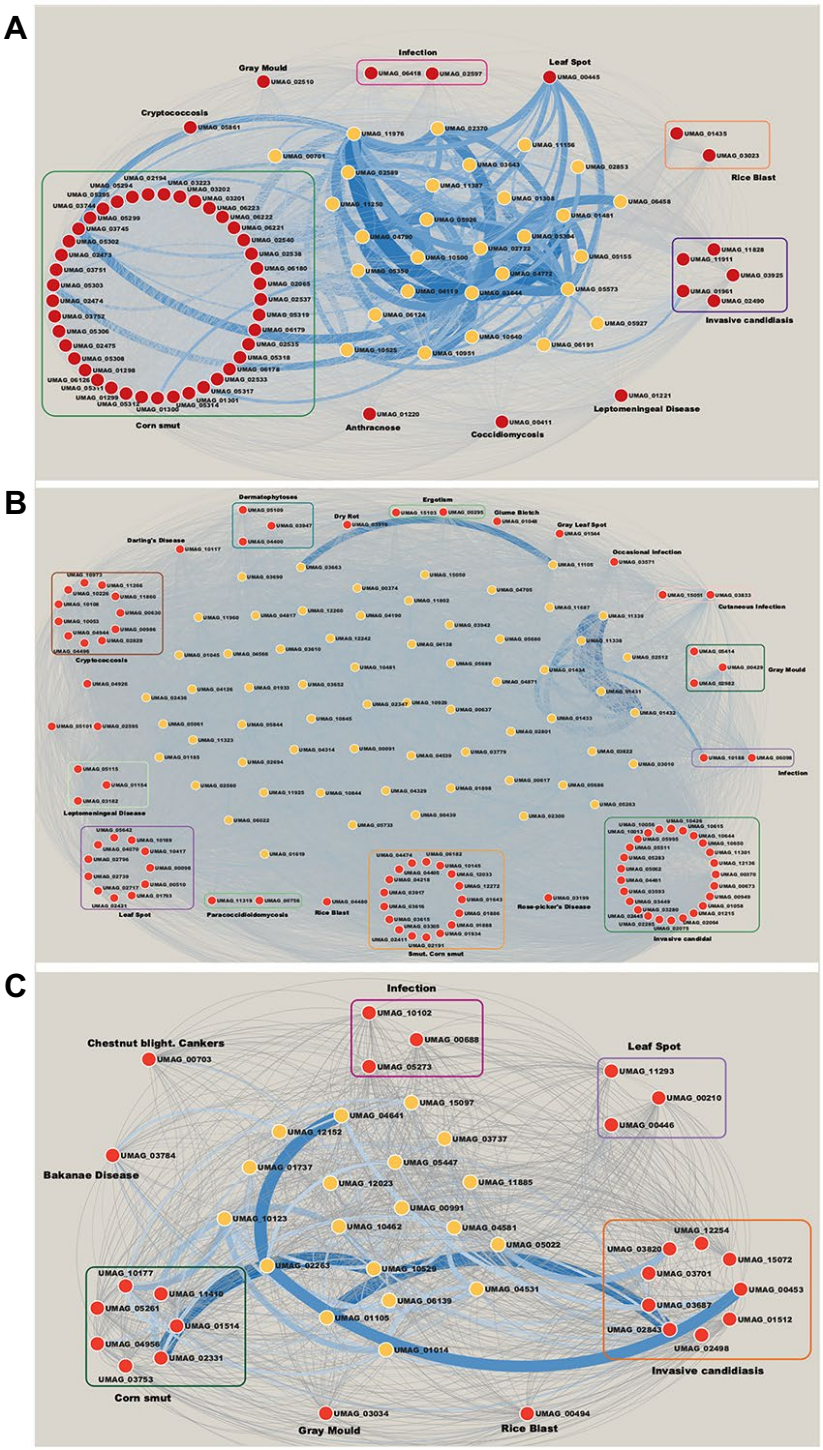
Networks of modules enrichment with virulence genes. **(A)** Brown module, **(B)** Magenta module, and **(C)** Yellow module. The virulence genes with their neighbor genes with the highest correlation were selected. The width of the edges is proportional to their correlation value. Red nodes are orthologs to virulence genes in Database of Virulence Factor in Fungal Pathogens.

In relation to modules enriched by virulence genes. We identified that the Brown module contains 56 ortholog genes related to virulence (Figure 5A). 41 genes are associated with Corn smut disease or Huitlacoche, which produces tumor-like galls on all meristematic tissues (Pataky and Snetselaar, 2006), and the remaining 15 genes are associated with other diseases (Figure 5A). Among the effector genes that are most co-expressed are those playing a critical role during the colonization of the plant, such as: UMAG_02538 regulated byRos1, the main regulator of spore formation (Tollot et al., 2016); UMAG_05303 that contains a DLNxxP motif that interacts with Topless/Topless proteins and leads to biotrophic susceptibility of plants (Darino et al., 2021); and the effector UMAG_03745 that can inhibit host defenses (Navarrete et al., 2021). These genes are mainly co-expressed with hypothetical genes such as: UMAG_06191, UMAG_10640, UMAG_10500, UMAG_11976, UMAG_03643, UMAG_01308, UMAG_01481, UMAG_04119, and UMAG_05573, which so they represent good candidates to study during *U. maydi*s infection.

Invasive candidiasis is the most represented disease in the Magenta module with 25 genes (Figure 5B). This disease causes pneumonia and meningitis in the lungs and brain, and is caused by *Candida albicans*, *C. glabrata*, *C. tropicalis*, *C. parapsilosis,* and *C. krusei* (Pappas et al., 2018). These human pathogenic fungi have the ability to switch between yeast and filamentous forms (dimorphic transition) associated with the infection process similar to what occurs between *U. maydis* and corn (Sánchez-Martínez and Pérez-Martín, 2001). In this way, the discovery of these virulence genes in the co-expressed modules indicates a molecular similarity between the two organisms. Besides, the most co-expressed genes in invasive candidiasis disease are: UMAG_02064, a putative H(+)-transporting V1 sector ATPase subunit F, involved in control of vacuolar pH in *Saccharomyces cerevisiae* (Poltermann et al., 2005); and UMAG_10650, homologous of TPS2 in *C. albicans*, related to the storage of trehalose and in the regulation of autophagy (Kim et al., 2021). These genes mostly co-expressed with UMAG_01434, UMAG_11338, UMAG_11338, UMAG_02512, UMAG_01431, UMAG_11339, and UMAG_01433 hypothetical genes (Figure 5B).

Finally, the Yellow module contains 26 genes related to virulence. It is the most diverse module in relation to associated diseases with nine genes related to invasive candidiasis, seven to corn smut disease, three to leaf spot disease, and 3 to infection, among others (Figure 5C). Some related genes are: UMAG_02843, homologous to KEX2, a subtilisin-like protease in *C. albicans.* Mutation of this gene has shown inability to form hyphae and diminishes the virulence of *C. albicans* (Newport and Agabian, 1997; Newport et al., 2003). And UMAG_00453, homologous of VPS34, a phosphatidylinositol 3-kinase, involved in cellular processes, such as mTOR signaling, trimeric G-protein signaling to MAPK in yeast, and autophagy (Backer, 2008; Carracedo and Pandolfi, 2008). The hypothetical genes UMAG_02263, UMAG_01105, UMAG_10529 and UMAG_10123, are mainly co-expressed with these genes (Figure 5C).

To determine a possible function of these hypothetical genes, their protein sequences were analyzed with InterProScan (Blum et al., 2021), to identify conserved structural domains (Table 3). We identified proteins with kinase-like domain, as UMAG_10529 and UMAG_10123, which has been associated to proteins that participate in cellular processes such as proliferation, adhesion, motility, and apoptosis, amount others (Engh and Bossemeyer, 2002); the P-loop containing nucleoside triphosphate hydrolase domain, in UMAG_01105 and UMAG_01431, present in proteins related to programmed cell death, disease, and stress response in different organisms (Leipe et al., 2004); as well as, two genes with protease domains: UMAG_02263 with Peptidase C15, pyroglutamyl peptidase I-like superfamily and UMAG_01433 with ClpP/crotonase-like domain superfamily.

**Table 3:** Probable targets most co-expressed with virulence genes.

Probable target	Co-expressed virulence genes	Related disease	Domain description	Module
UMAG_10951	UMAG_02538	Corn smut	AMP-binding enzyme, C-terminal domain superfamily	Brown
UMAG_10525	UMAG_02538	Corn smut	Cytoplasmic domain	Brown
UMAG_10525	UMAG_02538	Corn smut	Cytoplasmic domain, transmembrane	Brown
UMAG_06191	UMAG_01961	Invasive candidiasis	D-isomer specific 2-hydroxyacid dehydrogenase, NAD-binding domain	Brown
UMAG_03643	UMAG_02538	Corn smut	Nucleotide-diphospho-sugar transferases	Brown
UMAG_10640	UMAG_02538	Corn smut	RlpA-like domain superfamily	Brown
UMAG_10500	UMAG_02538	Corn smut	Zn(2)-C6 fungal-type DNA-binding domain superfamily; Thiamine repressible genes regulatory protein Thi5	Brown
UMAG_01434	UMAG_10188	Infection	Acetyl-CoA synthetase-like, CoA-dependent acyltransferases	Magenta
UMAG_11338	UMAG_10189	Infection	FAD/NAD(P)-binding domain superfamily	Magenta
UMAG_01432	UMAG_10190	Infection	Acyl-CoA N-acyltransferase	Magenta
UMAG_02512	UMAG_10191	Infection	Phosphofructokinase superfamily	Magenta
UMAG_01431	UMAG_10192	Infection	ABC transporter type 1, transmembrane domain superfamily; P-loop containing nucleoside triphosphate hydrolase	Magenta
UMAG_11339	UMAG_10193	Infection	MFS transporter superfamily	Magenta
UMAG_01433	UMAG_10194	Infection	Enoyl-CoA hydratase/isomerase; ClpP/crotonase-like domain superfamily	Magenta
UMAG_02263	UMAG_00453	Invasive candidal disease	Peptidase C15, pyroglutamyl peptidase I-like	Yellow
UMAG_01105	UMAG_02843	Invasive candidal disease	P-loop containing nucleoside triphosphate hydrolases superfamily; ABC transporter transmembrane region superfamily	Yellow
UMAG_10529	UMAG_02331	Smit Corn smut	Protein kinase-like domain superfamily	Yellow
UMAG_10123	UMAG_02331, UMAG_10177	Smit Corn smut	Protein kinase-like domain superfamily	Yellow

Several proteases have been identified as causative agents of fungal diseases. Growth, formation of infectious structures, cell wall degradation, proteolysis of proteins involved in pathogenesis and acting as initiators of defense responses (Chandrasekaran et al., 2016). The proteases in *U. maydis* are related to different functions such as virulence proteins, dimorphism, pathogenesis and autophagy (Mueller et al., 2008; Soberanes-Gutiérrez et al., 2015; Ökmen et al., 2018; Soberanes-Gutiérrez et al., 2019). Finally, we identified another 60 genes with conserved structural domains, in addition we identified a set of highly co-expressed genes that do not have conserved domains (Supplementary Table S6).

## Conclusion

In this work, we constructed a gene co-expression network for the pathogenic fungus *U. maydis* from gene expression data. This network was clustered into modules that are associated with different general biological functions, such as riboprotein complexes, and others more specific to the cell cycle or autophagy.

In addition, these modules were analyzed in terms of regulation and virulence. We identified that all the modules have at least one TF associated, which is consistent with other studies. In this regard, we found that the TFs associated with the enriched modules belong to the superfamilies of Nucleic acid-binding proteins, Winged helix DNA-binding, and Zn2/Cys6 DNA-binding. These proteins regulate a large number of cellular functions, ranging from cell division, metabolic processes to stress response and virulence.

On the other hand, there are modules enriched with virulence genes, mostly related to corn smut disease. These genes are effectors previously described in *U. maydis*. However, orthologs of other diseases tend to be identified in the modules, as in the case of Invasive candidiasis. The discovery of these virulence genes indicates that there is a molecular similarity between the pathogenic lifestyles. These similarities suggest that findings discovered in *U. maydis* might yield insights into virulence mechanisms of other pathogenic fungi. Finally, a large number of hypothetical genes were identified which are co-expressed with virulence-associated genes, making them excellent targets for possible experimental studies.

## Data availability statement

The original contributions presented in the study are included in the article/Supplementary material, further inquiries can be directed to the corresponding author.

## Author contributions

AC-J and CS-G carried out data acquisition, experiments, and analysis. EG-V and EP-R drafted the manuscript and provided scientific advice and contributed to results interpretations. All authors contributed to the article and approved the submitted version.

## Funding

This work was supported by the DGAPA of the Universidad Nacional Autónoma de México (IA201221, IA207423, IN-209620, and IN-220523), and CONACYT (320012).

## Conflict of interest

The authors declare that the research was conducted in the absence of any commercial or financial relationships that could be construed as a potential conflict of interest.

## Publisher’s note

All claims expressed in this article are solely those of the authors and do not necessarily represent those of their affiliated organizations, or those of the publisher, the editors and the reviewers. Any product that may be evaluated in this article, or claim that may be made by its manufacturer, is not guaranteed or endorsed by the publisher.

## Footnotes

^1^ http://david.abcc.ncifcrf.gov/

^2^ https://www.python.org/

## References

[ref1] Agrios G. N. (2005). “Environmental effects on the development of infectious plant disease” in Plant Pathology. ed. Cambridge M. A.. 5th ed (USA: Elsevier Academic Press), 251–265. doi: 10.1016/B978-0-08-047378-9.50013-0

[ref2] Araki H. Ropp P. A. Johnson A. L. Johnston L. H. Morrison A. Sugino A. (1992). DNA polymerase II, the probable homolog of mammalian DNA polymerase epsilon, replicates chromosomal DNA in the yeast *Saccharomyces cerevisiae*. EMBO J. 11, 733–740. doi: 10.1002/j.1460-2075.1992.tb05106.x, PMID: 1537345 PMC556506

[ref3] Backer J. M. (2008). The regulation and function of class III PI3Ks: novel roles for Vps34. Biochem. J. 410, 1–17. doi: 10.1042/BJ20071427, PMID: 18215151

[ref4] Bakhtiarizadeh M. R. Hosseinpour B. Shahhoseini M. Korte A. Gifani P. (2018). Weighted gene co-expression network analysis of endometriosis and identification of functional modules associated with its main hallmarks. Front. Genet. 9:453. doi: 10.3389/fgene.2018.00453, PMID: 30369943 PMC6194152

[ref5] Bakkeren G. Kämper J. Schirawski J. (2008). Sex in smut fungi: structure, function and evolution of mating-type complexes. Fungal Genet. Biol. 45, S15–S21. doi: 10.1016/j.fgb.2008.04.005, PMID: 18501648

[ref6] Basse C. W. Steinberg G. (2004). *Ustilago maydis*, model system for analysis of the molecular basis of fungal pathogenicity. Mol. Plant Pathol. 5, 83–92. doi: 10.1111/j.1364-3703.2004.00210.x, PMID: 20565585

[ref7] Blum M. Chang H. Y. Chuguransky S. Grego T. Kandasaamy S. Mitchell A.. (2021). The InterPro protein families and domains database: 20 years on. Nucleic Acids Res. 49, D344–D354. doi: 10.1093/nar/gkaa977, PMID: 33156333 PMC7778928

[ref8] Brefort T. Doehlemann G. Mendoza-Mendoza A. Reissmann S. Djamei A. Kahmann R. (2009). *Ustilago maydis* as a pathogen. Annu. Rev. Phytopathol. 47, 423–445. doi: 10.1146/annurev-phyto-080508-081923, PMID: 19400641

[ref9] Carracedo A. Pandolfi P. P. (2008). The PTEN–PI3K pathway: of feedbacks and cross-talks. Oncogene 27, 5527–5541. doi: 10.1038/onc.2008.247, PMID: 18794886

[ref10] Cerqueira G. C. Arnaud M. B. Inglis D. O. Skrzypek M. S. Binkley G. Simison M.. (2014). The *aspergillus* genome database: multispecies curation and incorporation of RNA-Seq data to improve structural gene annotations. Nucleic Acids Res. 42, D705–D710. doi: 10.1093/nar/gkt1029, PMID: 24194595 PMC3965050

[ref11] Chandrasekaran M. Thangavelu B. Chun S. C. Sathiyabama M. (2016). Proteases from phytopathogenic fungi and their importance in phytopathogenicity. J. Gen. Plant Pathol. 82, 233–239. doi: 10.1007/s10327-016-0672-9, PMID: 36335652

[ref12] Costa-Silva J. Domingues D. Lopes F. M. (2017). RNA-Seq differential expression analysis: an extended review and a software tool. PLoS One 12:e0190152. doi: 10.1371/journal.pone.0190152, PMID: 29267363 PMC5739479

[ref13] Darino M. Chia K. S. Marques J. Aleksza D. Soto-Jiménez L. M. Saado I.. (2021). *Ustilago maydis* effector Jsi1 interacts with topless corepressor, hijacking plant jasmonate/ethylene signaling. New Phytol. 229, 3393–3407. doi: 10.1111/nph.17116, PMID: 33247447 PMC8126959

[ref14] Dean R. Van Kan J. A. Pretorius Z. A. Hammond-Kosack K. E. Di Pietro A. Spanu P. D.. (2012). The top 10 fungal pathogens in molecular plant pathology. Mol. Plant Pathol. 13, 414–430. doi: 10.1111/j.1364-3703.2011.00783.x, PMID: 22471698 PMC6638784

[ref15] Eichhorn H. Lessing F. Winterberg B. Schirawski J. Kämper J. Müller P.. (2006). A ferroxidation/permeation iron uptake system is required for virulence in *Ustilago maydis*. Plant Cell 18, 3332–3345. doi: 10.1105/tpc.106.043588, PMID: 17138696 PMC1693961

[ref16] Elías-Villalobos A. Fernández-Álvarez A. Ibeas J. I. (2011). The general transcriptional repressor Tup1 is required for dimorphism and virulence in a fungal plant pathogen. PLoS Pathog. 7:e1002235. doi: 10.1371/journal.ppat.1002235, PMID: 21909277 PMC3164652

[ref17] Engh R. A. Bossemeyer D. (2002). Structural aspects of protein kinase control—role of conformational flexibility. Pharmacol. Ther. 93, 99–111. doi: 10.1016/S0163-7258(02)00180-8, PMID: 12191603

[ref18] Fang W. St. Leger R. J. (2010). RNA binding proteins mediate the ability of a fungus to adapt to the cold. Environ. Microbiol. 12, 810–820. doi: 10.1111/j.1462-2920.2009.02127.x, PMID: 20050869

[ref19] Ferris A. C. Walbot V. (2020). Understanding *Ustilago maydis* infection of multiple maize organs. J. Fungi 7:8. doi: 10.3390/jof7010008, PMID: 33375485 PMC7823922

[ref20] Gajiwala K. S. Burley S. K. (2000). Winged helix proteins. Curr. Opin. Struct. Biol. 10, 110–116. doi: 10.1016/S0959-440X(99)00057-3, PMID: 10679470

[ref21] Heimel K. Scherer M. Schuler D. Kämper J. (2010A). The *Ustilago maydis* Clp1 protein orchestrates pheromone and b-dependent signaling pathways to coordinate the cell cycle and pathogenic development. Plant Cell 22, 2908–2922. doi: 10.1105/tpc.110.076265, PMID: 20729384 PMC2947178

[ref22] Heimel K. Scherer M. Vranes M. Wahl R. Pothiratana C. Schuler D.. (2010). The transcription factor Rbf1 is the master regulator for b-mating type controlled pathogenic development in *Ustilago maydis*. PLoS Pathog. 6:e1001035. doi: 10.1371/journal.ppat.1001035, PMID: 20700446 PMC2916880

[ref23] Horst R. J. Zeh C. Saur A. Sonnewald S. Sonnewald U. Voll L. M. (2012). The *Ustilago maydis* Nit2 homolog regulates nitrogen utilization and is required for efficient induction of filamentous growth. Eukaryot. Cell 11, 368–380. doi: 10.1128/EC.05191-11, PMID: 22247264 PMC3294441

[ref24] Horvath S. (2011). Weighted network analysis: Applications in genomics and systems biology. New York, NY: Springer Science & Business Media.

[ref25] Huang D. W. Sherman B. T. Lempicki R. A. (2009). Systematic and integrative analysis of large gene lists using DAVID bioinformatics resources. Nat. Protoc. 4, 44–57. doi: 10.1038/nprot.2008.211, PMID: 19131956

[ref26] Junker B. H. Schreiber F. (eds.) (2008). Analysis of Biological Networks. Vol. 2. Hoboken, NJ: Wiley-Interscience. 31–59.

[ref27] Kämper J. (2004). A PCR-based system for highly efficient generation of gene replacement mutants in *Ustilago maydis*. Mol. Gen. Genomics. 271, 103–110. doi: 10.1007/s00438-003-0962-8, PMID: 14673645

[ref28] Kim H. J. (2014). Exploitation of reactive oxygen species by fungi: roles in host-fungus interaction and fungal development. J. Microbiol. Biotechnol. 24, 1455–1463. doi: 10.4014/jmb.1407.07072, PMID: 25152060

[ref29] Kim B. Lee Y. Choi H. Huh W. K. (2021). The trehalose-6-phosphate phosphatase Tps2 regulates ATG8 transcription and autophagy in *Saccharomyces cerevisiae*. Autophagy 17, 1013–1027. doi: 10.1080/15548627.2020.1746592, PMID: 32240040 PMC8078664

[ref30] Kitano H. (2002). Systems biology: a brief overview. Science 295, 1662–1664. doi: 10.1126/science.1069492, PMID: 11872829

[ref31] Knox B. P. Keller N. P. (2015). “Key players in the regulation of fungal secondary metabolism” in Biosynthesis and Molecular Genetics of Fungal Secondary Metabolites (New York, NY: Springer), 13–28. doi: 10.1007/978-1-4939-2531-5_2

[ref32] Langfelder P. Holvarth S. (2008). WGCNA: an R package for weighted correlation network analysis. BMC Bioinform. 9:559. doi: 10.1186/1471-2105-9-559 PMC2631488 19114008

[ref33] Langfelder P. Mischel P. S. Horvath S. (2013). When is hub gene selection better than standard meta-analysis? PLoS One 8:e61505. doi: 10.1371/journal.pone.0061505, PMID: 23613865 PMC3629234

[ref34] Lanver D. Berndt P. Tollot M. Naik V. Vranes M. Warmann T.. (2014). Plant surface cues prime *Ustilago maydis* for biotrophic development. PLoS Pathog. 10:e1004272. doi: 10.1371/journal.ppat.1004272, PMID: 25033195 PMC4102580

[ref35] Lanver D. Müller A. N. Happel P. Schweizer G. Haas F. B. Franitza M.. (2018). The biotrophic development of *Ustilago maydis* studied by RNA-Seq analysis. Plant Cell 30, 300–323. doi: 10.1105/tpc.17.00764, PMID: 29371439 PMC5868686

[ref36] Lechner M. Findeiß S. Steiner L. Marz M. Stadler P. F. Prohaska S. J. (2011). Proteinortho: detection of (co-) orthologs in large-scale analysis. BMC Bioinform. 12, 1–9. doi: 10.1186/1471-2105-12-124 PMC3114741 21526987

[ref37] Leipe D. D. Koonin E. V. Aravind L. (2004). STAND, a class of P-loop NTPases including animal and plant regulators of programmed cell death: multiple, complex domain architectures, unusual phyletic patterns, and evolution by horizontal gene transfer. J. Mol. Biol. 343, 1–28. doi: 10.1016/j.jmb.2004.08.023, PMID: 15381417

[ref38] Liang Y. F. Long Z. X. Zhang Y. J. Luo C. Y. Yan L. T. Gao W. Y.. (2021). The chemical mechanisms of the enzymes in the branched-chain amino acids biosynthetic pathway and their applications. Biochimie 184, 72–87. doi: 10.1016/j.biochi.2021.02.008, PMID: 33607240

[ref39] Liu W. Li L. Long X. You W. Zhong Y. Wang M.. (2018). Construction and analysis of gene co-expression networks in *Escherichia coli*. Cells 7:19. doi: 10.3390/cells7030019, PMID: 29518040 PMC5870351

[ref40] Lu T. Yao B. Zhang C. (2012). DFVF: database of fungal virulence factors. Database 2012:bas032. doi: 10.1093/database/bas032, PMID: 23092926 PMC3478563

[ref41] Lumsden T. Bentley A. A. Beutler W. Ghosh A. Galkin O. Komar A. A. (2010). Yeast strains with N-terminally truncated ribosomal protein S5: implications for the evolution, structure and function of the Rps5/Rps7 proteins. Nucleic Acids Res. 38, 1261–1272. doi: 10.1093/nar/gkp1113, PMID: 19969550 PMC2831326

[ref42] MacPherson S. Larochelle M. Turcotte B. (2006). A fungal family of transcriptional regulators: the zinc cluster proteins. Microbiol. Mol. Biol. Rev. MMBR. 70, 583–604. doi: 10.1128/MMBR.00015-06, PMID: 16959962 PMC1594591

[ref43] Martínez-Soto D. Ruiz-Herrera J. (2013). Transcriptomic analysis of the dimorphic transition of *Ustilago maydis* induced in vitro by a change in pH. Fungal Genet. Biol. 58–59, 116–125. doi: 10.1016/j.fgb.2013.08.011, PMID: 23994320

[ref44] Matei A. Ernst C. Günl M. Thiele B. Altmüller J. Walbot V.. (2018). How to make a tumour: cell type specific dissection of *Ustilago maydis* induced tumour development in maize leaves. New Phytol. 217, 1681–1695. doi: 10.1111/nph.14960, PMID: 29314018

[ref45] Medina I. R. Lubovac-Pilav Z. (2016). Gene co-expression network analysis for identifying modules and functionally enriched pathways in type 1 diabetes. PLoS One 11:e0156006. doi: 10.1371/journal.pone.0156006, PMID: 27257970 PMC4892488

[ref46] Molina L. Kahmann R. (2007). An Ustilago maydis gene involved in H2O2 detoxification is required for virulence. Plant Cell 19, 2293–2309. doi: 10.1105/tpc.107.052332, PMID: 17616735 PMC1955693

[ref47] Mueller A. J. Canty-Laird E. G. Clegg P. D. Tew S. R. (2017). Cross-species gene modules emerge from a systems biology approach to osteoarthritis. NPJ Syst. Biol. Appl. 3:13. doi: 10.1038/s41540-017-0014-3, PMID: 28649440 PMC5460168

[ref48] Mueller O. Kahmann R. Aguilar G. Trejo-Aguilar B. Wu A. de Vries R. P. (2008). The secretome of the maize pathogen *Ustilago maydis*. Fungal Genet. Biol. 45, S63–S70. doi: 10.1016/j.fgb.2008.03.012, PMID: 18456523

[ref49] Nair U. Yen W. L. Mari M. Cao Y. Xie Z. Baba M.. (2012). A role for Atg8–PE deconjugation in autophagosome biogenesis. Autophagy 8, 780–793. doi: 10.4161/auto.19385, PMID: 22622160 PMC3378420

[ref50] Navarrete F. Grujic N. Stirnberg A. Saado I. Aleksza D. Gallei M.. (2021). The Pleiades are a cluster of fungal effectors that inhibit host defenses. PLoS Pathog. 17:e1009641. doi: 10.1371/journal.ppat.1009641, PMID: 34166468 PMC8224859

[ref51] Newport G. Agabian N. (1997). KEX2 influences *Candida albicans* proteinase secretion and hyphal formation. J. Biol. Chem. 272, 28954–28961. doi: 10.1074/jbc.272.46.28954, PMID: 9360967

[ref52] Newport G. Kuo A. Flattery A. Gill C. Blake J. J. Kurtz M. B.. (2003). Inactivation of Kex2p diminishes the virulence of *Candida albicans*. J. Biol. Chem. 278, 1713–1720. doi: 10.1074/jbc.M209713200, PMID: 12419804

[ref53] Obokata J. Ohme M. Hayashida N. (1991). Nucleotide sequence of a cDNA clone encoding a putative glycine-rich protein of 19.7 kDa in *Nicotiana sylvestris*. Plant Mol. Biol. 17, 953–955. doi: 10.1007/BF00037080, PMID: 1912512

[ref54] Ökmen B. Kemmerich B. Hilbig D. Wemhöner R. Aschenbroich J. Perrar A.. (2018). Dual function of a secreted fungalysin metalloprotease in *Ustilago maydis*. New Phytol. 220, 249–261. doi: 10.1111/nph.15265, PMID: 29916208

[ref55] Olicón-Hernández D. R. Araiza-Villanueva M. G. Pardo J. P. Aranda E. Guerra-Sánchez G. (2019). New insights of *Ustilago maydis* as yeast model for genetic and biotechnological research: a review. Curr. Microbiol. 76, 917–926. doi: 10.1007/s00284-019-01629-4, PMID: 30689003

[ref56] Pappas P. G. Lionakis M. S. Arendrup M. C. Ostrosky-Zeichner L. Kullberg B. J. (2018). Invasive candidiasis. Nat. Rev. Dis. Primers 4, 1–20. doi: 10.1038/nrdp.2018.26 29749387

[ref57] Pataky J. K. Snetselaar K. M. (2006). Common smut of corn. Plant Health Instructor. 10. doi: 10.1094/PHI-I-2006-0927-01, PMID: 36161006

[ref58] Poltermann S. Nguyen M. Günther J. Wendland J. Härtl A. Künkel W.. (2005). The putative vacuolar ATPase subunit Vma7p of *Candida albicans* is involved in vacuole acidification, hyphal development and virulence. Microbiology 151, 1645–1655. doi: 10.1099/mic.0.27505-0, PMID: 15870472

[ref59] Ritchie M. E. Phipson B. Wu D. Hu Y. Law C. W. Shi W.. (2015). Limma powers differential expression analyses for RNA-sequencing and microarray studies. Nucleic Acids Res. 43:e47. doi: 10.1093/nar/gkv007, PMID: 25605792 PMC4402510

[ref60] Rodríguez-Kessler M. Baeza-Montañez L. García-Pedrajas M. D. Tapia-Moreno A. Gold S. Jiménez-Bremont J. F.. (2012). Isolation of UmRrm75, a gene involved in dimorphism and virulence of *Ustilago maydis*. Microbiol. Res. 167, 270–282. doi: 10.1016/j.micres.2011.10.007, PMID: 22154329

[ref61] Sánchez-Martínez C. Pérez-Martín J. (2001). Dimorphism in fungal pathogens: *Candida albicans* and *Ustilago maydis* similar inputs, different outputs. Curr. Opin. Microbiol. 4, 214–221. doi: 10.1016/S1369-5274(00)00191-0, PMID: 11282479

[ref62] Sastry A. V. Gao Y. Szubin R. Hefner Y. Xu S. Kim D.. (2019). The *Escherichia coli* transcriptome mostly consists of independently regulated modules. Nat. Commun. 10, 1–14. doi: 10.1038/s41467-019-13483-w 31797920 PMC6892915

[ref63] Sato N. (1995). A family of cold-regulated RNA-binding protein genes in the cyanobacterium *Anabaena variabilis* M3. Nucleic Acids Res. 23, 2161–2167. doi: 10.1093/nar/23.12.2161, PMID: 7541909 PMC307003

[ref64] Saville B. J. Donaldson M. E. Doyle C. E. (2012). Investigating host induced meiosis in a fungal plant pathogen. Meiosis Molecular Mech. Cytogenet. Divers. 22, 411–460. doi: 10.5772/30032

[ref65] Scherer M. Heimel K. Starke V. Kämper J. (2016). The Clp1 protein is required for clamp formation and pathogenic development of *Ustilago maydis*. Plant Cell 18, 2388–2401. doi: 10.1105/tpc.106.043521 PMC1560919 16920779

[ref66] Schuler D. Wahl R. Wippel K. Vranes M. Münsterkötter M. Sauer N.. (2015). Hxt1, a monosaccharide transporter and sensor required for virulence of the maize pathogen *Ustilago maydis*. New Phytol. 206, 1086–1100. doi: 10.1111/nph.13314, PMID: 25678342

[ref67] Schuster M. Schweizer G. Reissmann S. Kahmann R. (2016). Genome editing in *Ustilago maydis* using the CRISPR-Cas system. Fungal Genet. Biol. 89, 3–9. doi: 10.1016/j.fgb.2015.09.001, PMID: 26365384

[ref68] Sharifulin D. Babaylova E. Kossinova O. Bartuli Y. Graifer D. Karpova G. (2013). Ribosomal protein S5e is implicated in translation initiation through its interaction with the N-terminal domain of initiation factor eIF2α. Chembiochem 14, 2136–2143. doi: 10.1002/cbic.201300318, PMID: 24106102

[ref69] Shelest E. (2008). Transcription factors in fungi. FEMS Microbiol. Lett. 286, 145–151. doi: 10.1111/j.1574-6968.2008.01293.x, PMID: 18789126

[ref70] Skibbe D. S. Doehlemann G. Fernandes J. Walbot V. (2010). Maize tumors caused by *Ustilago maydis* require organ-specific genes in host and pathogen. Science 328, 89–92. doi: 10.1126/science.1185775, PMID: 20360107

[ref71] Snetselaar K. McCann M. (2017). *Ustilago maydis*, the corn smut fungus, has an unusual diploid mitotic stage. Mycologia 109, 140–152. doi: 10.1080/00275514.2016.1274597, PMID: 28402788

[ref72] Soberanes-Gutiérrez C. V. Juárez-Montiel M. Olguín-Rodríguez O. Hernández-Rodríguez C. Ruiz-Herrera J. Villa-Tanaca L. (2015). The pep4 gene encoding proteinase a is involved in dimorphism and pathogenesis of *Ustilago maydis*. Mol. Plant Pathol. 16, 837–846. doi: 10.1111/mpp.12240, PMID: 25597948 PMC6638482

[ref73] Soberanes-Gutiérrez C. V. Pérez-Rueda E. Ruíz-Herrera J. Galán-Vásquez E. (2021). Identifying genes devoted to the cell death process in the gene regulatory network of *Ustilago maydis*. Front. Microbiol. 12:1321. doi: 10.3389/fmicb.2021.680290 PMC8175908 34093501

[ref74] Soberanes-Gutiérrez C. V. Vázquez-Carrada M. Lopez-Villegas E. O. Vega-Arreguín J. C. Villa-Tanaca L. Ruiz-Herrera J. (2019). Autophagosomes accumulation in the vacuoles of the fungus *Ustilago maydis* and the role of proteases in their digestion. FEMS Microbiol. Lett. 366:fnz108. doi: 10.1093/femsle/fnz108, PMID: 31183499

[ref75] Song L. Langfelder P. Horvath S. (2012). Comparison of co-expression measures: mutual information, correlation, and model based indices. BMC Bioinform. 13:328. doi: 10.1186/1471-2105-13-328, PMID: 23217028 PMC3586947

[ref76] Stark R. Grzelak M. Hadfield J. (2019). RNA sequencing: the teenage years. Nat. Rev. Genet. 20, 631–656. doi: 10.1038/s41576-019-0150-2, PMID: 31341269

[ref77] Storfie E. R. Saville B. J. (2021). Fungal pathogen emergence: investigations with an *Ustilago maydis*× *Sporisorium reilianum* hybrid. J. Fungi 7:672. doi: 10.3390/jof7080672, PMID: 34436211 PMC8400639

[ref78] Stuart J. Segal E. Koller D. Stuart K. (2003). A gene-coexpression network for global discovery of conserved genetic modules. Science 302, 249–255. doi: 10.1126/science.1087447, PMID: 12934013

[ref79] Takahashi T. Shimoi H. Ito K. (2001). Identification of genes required for growth under ethanol stress using transposon mutagenesis in *Saccharomyces cerevisiae*. Mol. Gen. Genomics. 265, 1112–1119. doi: 10.1007/s004380100510, PMID: 11523784

[ref80] Teertstra W. R. Krijgsheld P. Wösten H. A. (2011). Absence of repellents in *Ustilago maydis* induces genes encoding small secreted proteins. Antonie Van Leeuwenhoek 100, 219–229. doi: 10.1007/s10482-011-9581-2, PMID: 21626092 PMC3133707

[ref81] Tollot M. Assmann D. Becker C. Altmüller J. Dutheil J. Y. Wegner C. E.. (2016). The WOPR protein Ros1 is a master regulator of sporogenesis and late effector gene expression in the maize pathogen *Ustilago maydis*. PLoS Pathog. 12:e1005697. doi: 10.1371/journal.ppat.1005697, PMID: 27332891 PMC4917244

[ref82] Trewavas A. (2006). A brief history of systems biology. Plant Cell 18, 2420–2430. doi: 10.1105/tpc.106.042267, PMID: 17088606 PMC1626627

[ref83] Tsaparas P. Marino-Ramirez L. Bodenreider O. Koonin E. V. Jordan K. (2006). Global similarity and local divergence in human and mouse gene co-expression networks. BMC Evol. Biol. 6:70. doi: 10.1186/1471-2148-6-70, PMID: 16968540 PMC1601971

[ref84] van Dam S. Võsa U. van der Graaf A. Franke L. deMagalhães J. P. (2018). Gene co-expression analysis for functional classification and gene-disease predictions. Brief Bioinform. 19, 575–592. doi: 10.1093/bib/bbw139, PMID: 28077403 PMC6054162

[ref85] Van Noort V. Snel B. Huynen M. A. (2004). The yeast coexpression network has a small-world, scale-free architecture and can be explained by a simple model. EMBO Rep. 5, 280–284. doi: 10.1038/sj.embor.7400090, PMID: 14968131 PMC1299002

[ref86] Veri A. O. Robbins N. Cowen L. E. (2018). Regulation of the heat shock transcription factor Hsf1 in fungi: implications for temperature-dependent virulence traits. FEMS Yeast Res. 18:foy041. doi: 10.1093/femsyr/foy041, PMID: 29788061 PMC7190891

[ref87] Vollmeister E. Haag C. Zarnack K. Baumann S. König J. Mannhaupt G.. (2009). Tandem KH domains of Khd4 recognize AUACCC and are essential for regulation of morphology as well as pathogenicity in *Ustilago maydis*. RNA 15, 2206–2218. doi: 10.1261/rna.1817609, PMID: 19854870 PMC2779690

[ref88] Wang L. Berndt P. Xia X. Kahnt J. Kahmann R. (2011). A seven-WD40 protein related to human RACK1 regulates mating and virulence in *Ustilago maydis*. Mol. Microbiol. 81, 1484–1498. doi: 10.1111/j.1365-2958.2011.07783.x, PMID: 21815950

[ref89] Zhang B. Horvath S. (2005). A general framework for weighted gene co-expression network analysis. Stat. Appl. Genet. Mol. Biol. 4:Article17. doi: 10.2202/1544-6115.1128, PMID: 16646834

