# Supplementary material for: Construction and analysis of gene co-expression network in the pathogenic fungus Ustilago maydis
Source: Front Microbiol. 2022 Dec 7;13:1048694. doi: 10.3389/fmicb.2022.1048694 (PMC9767968; doi:10.3389/fmicb.2022.1048694)
Supplement: Supplementary file 7 [file Data_Sheet_1.PDF]

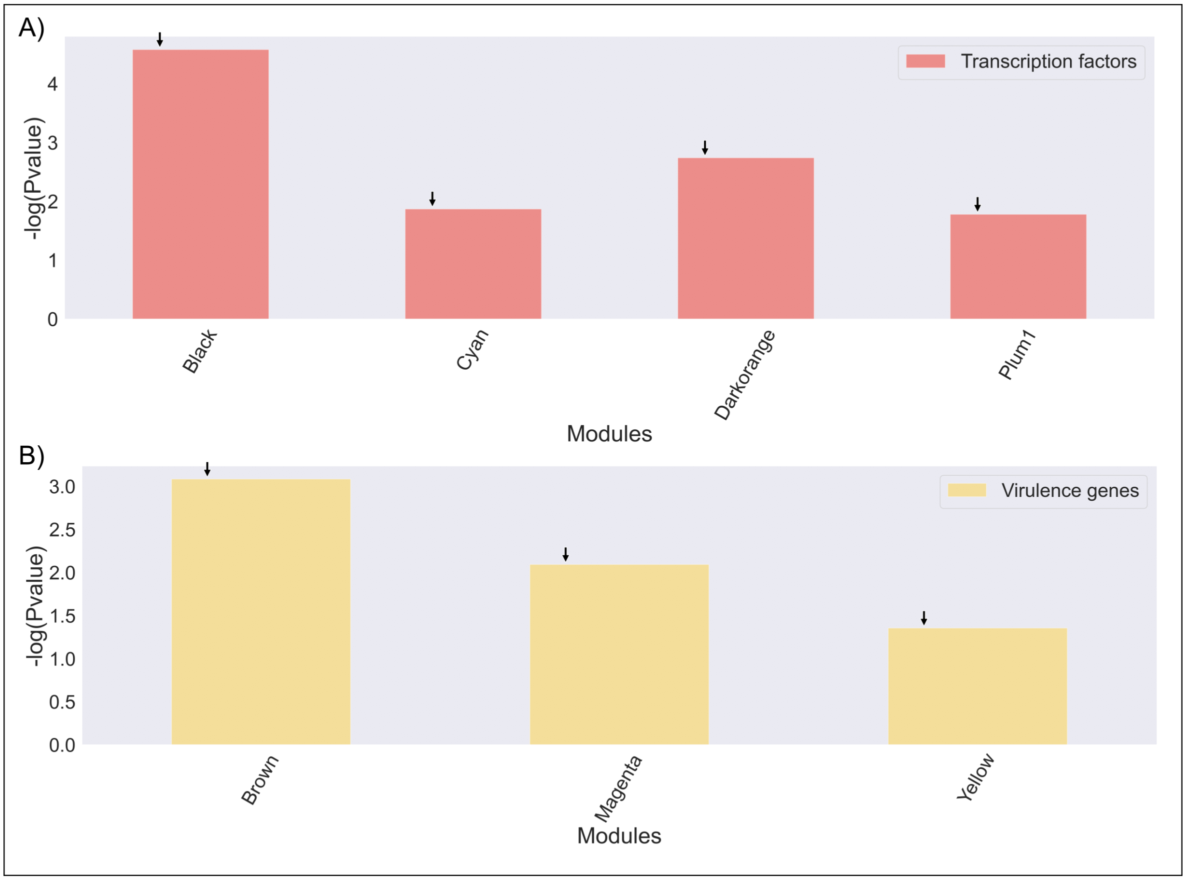

Figure S1. Enrichment of TFs and virulence genes. Modules with a -log10(P-value)>1.5 (corresponding to a P-value <0.05) were selected as enrichment and are indicated by an arrow on the bar.
