# Supplementary material for: Construction and analysis of gene co-expression network in the pathogenic fungus Ustilago maydis
Source: Front Microbiol. 2022 Dec 7;13:1048694. doi: 10.3389/fmicb.2022.1048694 (PMC9767968; doi:10.3389/fmicb.2022.1048694)
Supplement: Supplementary file 8 [file Data_Sheet_2.PDF]

```
#Pipeline to select modules of interest generated from WGCNA
import os
import pandas as pd
import numpy as np
import matplotlib.pyplot as plt
import seaborn as sns; sns.set()
from collections import Counter
import csv
from scipy.stats import hypergeom
import math

#Read the WGCNA module file
modules=pd.read_csv("ModulesUmaydis14Bicor.csv", sep='\t')

#Read the TFs file of U. maydis
TFs=pd.read_csv("TFUmaydis.txt", sep='\t')

#Read the virulence gene file
Virul=pd.read_csv("Virulence.txt", sep='\t')

#Association of modules to traits

modules["TFs"] = np.nan
modules['TFs'] = modules['TFs'].astype(object)
for row in modules.itertuples():
    for row1 in TFs.itertuples():
        if row.NameGen ==row1.Locus_tag:
            modules.loc[row.Index, 'TFs'] = row1.Pfam

modules["Virulence"] = np.nan
modules['Virulence'] = modules['Virulence'].astype(object)
for row in modules.itertuples():
    for row1 in Virul.itertuples():
        if row.NameGen ==row1.Locus_tag:
            modules.loc[row.Index, 'Virulence'] = row1.GeneVirulence


#Data preprocessing
d=pd.DataFrame()
for i in colores:
    df_filtered = modules[(modules.Mcolors == i)]
    tamaño=df_filtered.index.size
    TFsin=df_filtered['TFs'].count()
    Maps=(df_filtered['Mapas']!=0).sum()
    Virulence=df_filtered['Virulence'].count()
    d = d.append({'Modules': i, 'Size': tamaño, 'TFs': TFsin,'Maps':
Maps, 'Virulence': Virulence}, ignore_index=True)


datos=d[['Modules']]
```

```
datos["Size"] = np.nan
datos["TFs"] = np.nan
datos["Maps"] = np.nan
datos["Virulence"] = np.nan
datos["Unclassified"] = np.nan
for index1, row in d.iterrows():
    Tot=d['Size'][index1]
    TF=d['TFs'][index1]
    MP=d['Maps'][index1]
    Virulence=d['Virulence'][index1]
    SCla=Tot-(TF+MP+Virulence)

    datos.loc[index1,'Size']=Tot
    datos.loc[index1,'TFs']=TF
    datos.loc[index1,'Maps']=MP
    datos.loc[index1,'Virulence']=Virulence
    datos.loc[index1,'Unclassified']=SCla

#Module enrichment
d["Pvalue-TF"] = np.nan
N=6765 #total de genes
m=len(TFs.Locus_tag.unique()) #tf totales genoma
for index1, row in d.iterrows():
    x=d.TFs[index1] #tf modulo
    k=d.Size[index1] #Tamaño del modulo
    p = hypergeom.sf((x-1), N, k, m)
    d.loc[index1,'Pvalue-TF'] = p

d["Pvalue-Vr"] = np.nan
N=6765 #total de genes
m=len(Virul.Locus_tag.unique()) #Genes asociados a virulencia
for index1, row in d.iterrows():
    x=d.Virulence[index1] #virul en modulo
    k=d.Size[index1] #Tamaño del modulo
    p = hypergeom.sf((x-1), N, k, m)
    d.loc[index1,'Pvalue-Vr'] = p


d["TF_logP_value"] = np.nan
d["Map_logP_value"] = np.nan
d["Virulence_logP_value"] = np.nan
for index1, row in d.iterrows():
    d.loc[index1,'TF_logP_value'] = -(math.log10(d['Pvalue-TF']
[index1]))
    d.loc[index1,'Map_logP_value'] = -(math.log10(d['Pvalue-Mp']
[index1]))
    d.loc[index1,'Virulence_logP_value'] = -(math.log10(d['Pvalue-Vr']
[index1]))

dfil=pd.DataFrame( columns=['Modules','TF_logP_value','Map_logP_value'
```

```
, 'Virulence_logP_value'])
for index1, row in d.iterrows():
    if d['TF_logP_value'][index1] > 1.3 or d['Map_logP_value'][index1]
> 1.3 or d['Virulence_logP_value'][index1] > 1.3:
        dfil = dfil.append({'Modules':d['Modules'][index1],
'TF_logP_value':d['TF_logP_value']
[index1]  ,'Map_logP_value':d['Map_logP_value'][index1],
'Virulence_logP_value':d['Virulence_logP_value'][index1]},
ignore_index=True)


#Modules enriched with virulence genes

dfil=pd.DataFrame( columns=['Modules','Virulence_logP_value'])
for index1, row in d.iterrows():
    if d['Virulence_logP_value'][index1] > 1.3:
        dfil = dfil.append({'Modules':d['Modules'][index1],
'Virulence_logP_value':d['Virulence_logP_value'][index1]},
ignore_index=True)



ax=dfil.set_index('Modules')
[['Virulence_logP_value']].plot(kind='bar',
        figsize=(30,8), color=['#ffd343'], alpha=0.5,grid=0, fontsize
=30)
plt.xticks(rotation=60)
plt.ylabel("-log(Pvalue)", fontsize=35)
plt.xlabel("Modules", fontsize=35)

plt.legend(["Virulence genes"], fontsize=30);

for p in ax.patches:
    dat=p.get_height()
    if dat > 1.3:
        ax.annotate(str(''), (p.get_x()+.1, p.get_height()),
xytext=(0,30),
                    textcoords='offset points',
ha="center",va="center",
                    arrowprops=dict(facecolor='black', shrink=0.1))

plt.savefig('EnrichmentUmaydisVirulence.png',bbox_inches='tight',dpi=3
00)

#Modules enriched with TFs
dfil2=pd.DataFrame( columns=['Modules','TF_logP_value'])
for index1, row in d.iterrows():
    if d['TF_logP_value'][index1] > 1.3:
        dfil2 = dfil2.append({'Modules':d['Modules'][index1],
'TF_logP_value':d['TF_logP_value'][index1]}, ignore_index=True)
```

```
ax=dfil2.set_index('Modules')[['TF_logP_value']].plot(kind='bar',
        figsize=(30,8), color=['#EE3224'], alpha=0.5,grid=0, fontsize
=30)
plt.xticks(rotation=60)
plt.ylabel("-log(Pvalue)", fontsize=35)
plt.xlabel("Modules", fontsize=35)

plt.legend(["Transcription factors"], fontsize=30);

for p in ax.patches:
    dat=p.get_height()
    if dat > 1.3:
        ax.annotate(str(''), (p.get_x()+.1, p.get_height()),
xytext=(0,30),
                    textcoords='offset points',
ha="center",va="center",
                    arrowprops=dict(facecolor='black', shrink=0.1))

plt.savefig('EnrichmentUmaydisTFs.png',bbox_inches='tight',dpi=300)

#Superfamily association
x=1.3
modTFs=[]
for index1, row in d.iterrows():
    ds=d['TF_logP_value'][index1]
    if ds > x:
        ModSel1=d['Modules'][index1]
        modTFs.append(ModSel1)

Tf_filtered=modules[modules['Mcolors'].isin(modTFs)]
Tf_filtered.head()

Tf_filtered.to_csv("TFfiltradosUmaydis14bic.txt", sep='\t',
index=False, header=True)

Hmm_Name=Tf_filtered['TFs'].unique()
Hmm_Name = np.delete(Hmm_Name, 0, axis = 0)

dfHmm= pd.DataFrame(data=Hmm_Name.flatten(),  columns=["Hmm_Name"])

for i in modTFs:
    filt=Tf_filtered[Tf_filtered.Mcolors.isin([i])]
    dfHmm[i] = np.nan
    for index2, row2 in dfHmm.iterrows():
        dfHmm.at[index2,i] =
len(filt[filt.TFs.isin([row2["Hmm_Name"]])])

dfHmm1=dfHmm.set_index('Hmm_Name')
```

```
dfHmm1.fillna(0,inplace=True)
dfHmm1.head()

dfHmm1fil = dfHmm1[dfHmm1.sum(axis = 1) > 1]

TM = sns.clustermap(dfHmm1fil, cmap="BuPu",
                   linewidths=1, z_score=1, metric='cityblock',
figsize=[10,8],dendrogram_ratio=0.04)
ax = TM.ax_heatmap
x0, _y0, _w, _h = TM.cbar_pos
TM.ax_cbar.set_position([x0, 1, 0.05, 0.08])
TM.ax_cbar.tick_params(axis='x', length=30)
ax.set_ylabel("")
ax.figure.savefig("ClustTFs.png",bbox_inches='tight',dpi=300)
```
